# Impact of forage diversity on forage productivity, nutritive value, beef cattle performance, and enteric methane emissions

**DOI:** 10.1093/jas/skab326

**Published:** 2021-11-17

**Authors:** Logan R Thompson, Isabella C F Maciel, Patricia D R Rodrigues, Kim A Cassida, Jason E Rowntree

**Affiliations:** 1 Department of Animal Science, Michigan State University, East Lansing, MI 48824, USA; 2 Department of Plant, Soil and Microbial Sciences, Michigan State University, East Lansing, MI 48824, USA

**Keywords:** biodiversity, cattle, enteric methane, grazing

## Abstract

Greenhouse gas emissions from the beef industry are largely attributed to the grazing sector, specifically from beef cattle enteric methane emissions. Therefore, the study objective was to examine how forage diversity impacts forage productivity, nutritive value, animal performance, and enteric methane emissions. This study occurred over three consecutive grazing seasons (2018 to 2020) and compared two common Midwest grazing mixtures: 1) a simple, 50:50 alfalfa:orchardgrass mixture (**SIMP)** and 2) a botanically diverse, cool-season species mixture (**COMP**). Fifty-six steers and heifers were adapted to an Automated Head Chamber System (**AHCS**) each year (C-Lock Inc., Rapid City, SD) and stratified into treatment groups based on acclimation visitation. Each treatment consisted of four pastures, three 3.2-ha and one 1.6-ha, with eight and four animals each, respectively. Forage production was measured biweekly in pre- and postgrazed paddocks, and forage nutritive value was analyzed using near-infrared reflectance spectroscopy. Shrunk body weights were taken monthly to determine animal performance. Forage availability did not differ between treatments (*P* = 0.69) but tended lower in 2018 (*P* = 0.06; 2.40 t dry matter ha^−1^) than 2019 (2.92 t dry matter ha^−1^) and 2020 (*P* = 0.10; 2.81 t dry matter ha^−1^). Crude protein was significantly lower for COMP in 2018 compared with SIMP. Forage acid detergent fiber content was significantly lower for the COMP mixture (*P* = 0.02). The COMP treatment resulted higher dry matter digestibility (IVDMD48) in 2018 and 2019 compared with the SIMP treatment (*P* < 0.01). Animal performance did not differ between treatments (*P* > 0.50). There was a tendency for the COMP treatment to have lower enteric CH_4_ production on a g d^−1^ basis (*P* = 0.06), but no difference was observed on an emission intensity basis (g CH_4_ kg^−1^ gain; *P* = 0.56). These results would indicate that adoption of the complex forage mixture would not result in improved forage productivity, animal performance, or reduced emission intensity compared with the simple forage mixture.

## Introduction

The beef industry has come under scrutiny in recent decades due to its perceived contribution to global warming, with enteric methane (**CH**_**4**_) being the main contributor to the carbon footprint (Rotz et al., 2019). This has increased research to address mitigation options for both beef and dairy cattle to improve efficiency of production and reduce the carbon footprint (Boadi et al., 2004; Hristov et al., 2013; Thompson and Rowntree, 2020). Recent beef industry life-cycle assessments indicate that approximately 70% to 80% of the industry’s carbon emissions are from the grazing sectors, and predominately from cow–calf production, making mitigation in grazed environments crucial for overall enteric CH_4_ mitigation (Alemu et al., 2017; Rotz et al., 2019). Animals in grazed environments consume diets that are higher in fiber content than those in confinement operations and this high fiber diet drives greater enteric CH_4_ per unit of dry matter intake (**DMI**) with concurrently reduced animal performance (Thompson and Rowntree, 2020). These combine to result in greater total emissions and increased emissions per unit product (e.g., kg carcass weight; Stanley et al., 2018). Tested mitigation strategies include dietary lipid supplementation, vaccination against methanogens, utilizing forages with secondary compounds that reduce methanogenesis, improving forage digestibility, and animal health interventions, all of which have been reviewed extensively (Beauchemin et al., 2008; Hristov et al., 2013; Thompson and Rowntree, 2020; Zubieta et al., 2021). Mitigation strategies that have low barrier for entry and high potential adoption likelihood (i.e., inexpensive and easy to adopt) should be prioritized (Hristov et al., 2013). One such strategy would be the examination of different forage species bases to identify differences in animal performance, as well as monitor animal and soil greenhouse gas emissions. Proper forage species selection and grazing management can result in reduced enteric CH_4_ production by improving the digestibility of the diet (Beauchemin et al., 2008; Archimède et al., 2011).

A common pasture in the Midwestern United States is a binary alfalfa:orchardgrass mixture which has relatively high nutritive value. In a meta-analysis of enteric CH_4_ production of different forages, Archimède et al. (2011) indicated that cool-season forages typical of those growing in the Midwest United States produce lower enteric CH_4_ emissions compared with warm-season grasses per unit of DMI. Cool-season grasses utilize the C3 photosynthetic pathway and typically have less fiber content, decreased lignification and greater protein content (Barbehenn et al., 2004) than C4 grasses. Although there is inconsistency in research results, the inclusion of legumes in pastures may reduce enteric CH_4_ production through increased DMI and ruminal passage rate, reduced fiber content, improved animal performance and the presence of condensed tannins in some species, such as birdsfoot trefoil (Beauchemin et al., 2008; Hristov et al., 2013). However, the literature directly comparing enteric methane emissions and animal performance from a simple forage mixture to diverse forage mixtures is lacking (Alemu et al., 2019). Therefore, the objective of this experiment was to examine forage productivity, nutritive value, animal performance, and enteric CH_4_ emissions of two common Midwest grazing mixtures: a simple (**SIMP**) alfalfa:orchardgrass mixture and a complex (**COMP**) forage mixture. The hypothesis was that the COMP forage mixture would result in increased forage productivity, improved forage nutritive value, and a reduction in enteric methane emissions.

## Materials and Methods

The use of animals and procedures were approved by the Michigan State Animal Care and Use Committee (Protocol #02-18-019-00).

### Experimental design and pastures

**Figure 1.**
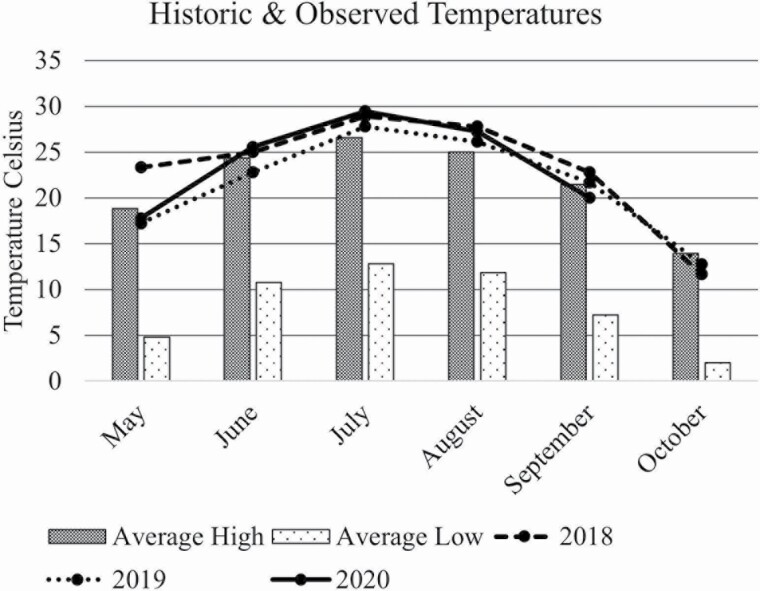
Lake City, MI 30-yr average temperature and observed temperatures.

**Figure 2.**
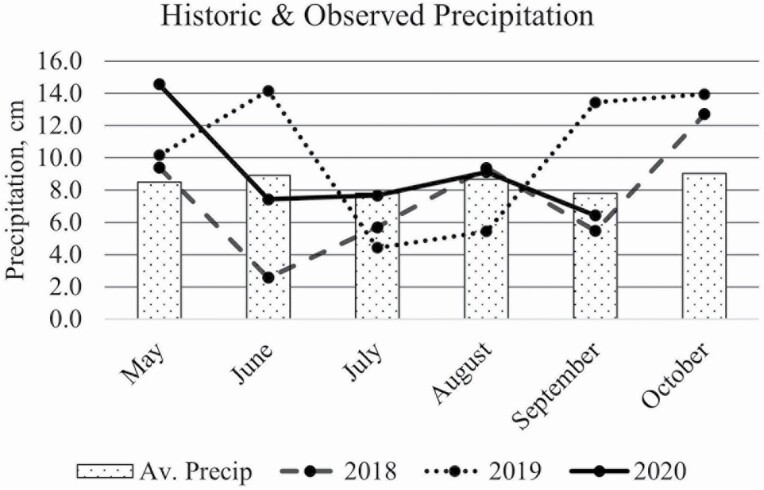
Lake City, MI 30-yr average precipitation and observed precipitation.

Experimental research pastures were located at the Michigan State University Lake City AgBioResearch Center (latitude: 44°18′N, longitude: 85°11′W; elevation 377 m; Appendix A) and the experiment consisted of three consecutive grazing seasons from 2018 through 2020. Onsite weather data were collected from a National Oceanic and Atmospheric Association weather station and are reported in Figures 1 and 2 (NOAA, 2020). Each treatment consisted of four experimental units: three 3.2-ha pastures and one 1.6-ha pasture, the unequal size due to flooding issues. Treatments were established in the fall of 2017 after termination of previous pasture using glyphosate and tillage. Previously, pastures were planted perennial grass or annual brassicas and no soil amendments were made before or after seeding experimental mixtures. Two pasture treatments were established: 1) a simple mixture and 2) a complex forage mixture. The SIMP pastures were seeded with 13.4 kg ha^−1^ alfalfa (*Medicago sativa* L. cv. ‘Ameristand 403T Plus’) and 4.2 kg ha^−1^ orchardgrass (*Dactylis glomerata* L. cv. ‘Tekapo’). The COMP mixture was seeded with 2.2 kg/ha each of ‘Ameristand 403T Plus’ alfalfa, red clover (*Trifolium pratense* L. cv. ‘Starfire’), ‘Tekapo’ orchardgrass, and timothy (*Phleum pratense* L. cv. ‘Climax’), 4.5 kg ha^−1^ each of birdsfoot trefoil (*Lotus corniculatus* L. cv. ‘Bull’), and meadow fescue (*Festuca pratensis Huds.* cv. ‘Pradel’), 1.1 kg ha^−1^ of chicory (*Cichorium intybus* L. cv. ‘Oasis’) and 0.2 kg ha^−1^ of white clover (*T. repens* L. cv. ‘Grasslands Kopu II’). Fertilizer was not applied to any of the treatment plots.

Forage was allocated using rotational management at a stocking density of 40 animals ha^−1^. The 3.2-ha pastures were assigned eight animals with sixteen 0.2-ha paddocks and 1.6-ha pastures were assigned four animals with eight 0.1-ha paddocks. In 2018, cattle in both treatments were rotated every 2 d for the duration of the grazing season. Two pastures within the COMP treatment were mowed to a 25-cm stubble height on June 17, 2018, to curb growth of reproductive stems of forage chicory. In 2019, cattle assigned to COMP treatments were rotated daily for the first 32 d (two full rotations) then slowed to 2-d moves with the SIMP treatment for the remainder of the year. This was done to increase grazing pressure on forage chicory. In 2020, the cattle in COMP pastures were rotated daily for the first 24 d, then all pastures were slowed to 3 d moves for the duration of the grazing.

### Animals

Annually, Red Angus steers and heifers (*n* = 56) were selected from the larger acclimation group and stratified into grazing groups based on acclimation visitation, to ensure that each replicate contained animals that would visit the Automated Head Chamber System (**ACHS**; GreenFeed, C-Lock Inc., Rapid City, SD) and randomly assigned to a pasture. Animals were placed in treatment pastures at day −4 to allow animals to adapt to their paddocks before beginning of sampling with the ACHS. Animals were offered ad libitum access to drinking water and commercially available free-choice mineral–vitamin mix (Hubbard Feeds, Mankato, MN).

Year 1 consisted of 56 steers (13-mo-old; BW = 318 ± 37.6 kg) and the grazing season started on June 1, 2018. Grazing duration was a total of 104 d in the experimental pastures. For 28 d (August 17 to September 14), all animals were removed due to low forage quantity (<600 kg ha^−1^ postgraze residuals) and managed in a single group until grazing could continue. These 28 d were not included in animal performance or enteric CH_4_ analysis. Before grazing (day −7) shrunk body weights were obtained from each animal after a 24 h shrink. Animals were reweighed monthly after 12 h shrinks and average daily gain (**ADG**) was determined via linear regression. When animals were returned to experimental pastures in September, 12-h shrunk weights were taken every 2 wk to improve precision of ADG estimates. Grazing was terminated on October 10, 2018.

Year 2 consisted of 48 Red Angus heifers and 8 steers (13-mo-old; BW = 283 ± 36.5 kg) selected from the larger acclimation group. Four steers were assigned to each of the 1.6-ha pastures based on acclimation visitation rate. Heifers were assigned experimental pastures based on breeding groups as dictated by the breeding goals of the research farm and randomly assigned to a pasture. One bull per pasture was turned out in heifer pastures from July 1 to July 29 for breeding. Shrunk body weights (12 h shrink) were taken before grazing onset (day −4) and monthly for the duration of the grazing season which began May 25, 2019 and ended on October 1, 2019.

Year 3 consisted of 48 Red Angus heifers and 8 steers (13-mo-old; BW = 267 ± 27.9) selected from the larger Automated Head Chamber System (**AHCS**) acclimation group. Again, four steers were stratified to each of the 1.6-ha pastures based on AHCS acclimation visitation. Heifers were selected based on ACHS acclimation then assigned to breeding groups dictated by farm breeding goals and randomly assigned to a pasture. Bulls were turned out from June 26 to July 24 for breeding. Shrunk body weights (12 h shrink) were taken before grazing onset (day −7) and again monthly for the duration of the grazing season which began on May 30, 2020 and ended on September 19, 2020.

### Gas production

Enteric CH_4_ and carbon dioxide (**CO**_**2**_) were estimated using the ACHS. Due to only one system being available, it was randomly assigned to one pasture, then rotated between treatment and paddocks every 2 wk for the duration of the grazing season so that each experimental replicate was monitored in each grazing season. The ACHS rotation pattern was randomized each year so that replicates were not analyzed at the same time each year. Animals were allowed a maximum of four visits each day, with six drops per visit, and a drop dispense interval of 30 s between each drop. This was done to ensure that animals remained in the partially enclosed chamber for a minimum of 3 min recommended by Velazco et al. (2016). Any visits less than 3 min were removed from the analysis. A minimum of 4 h was required between visits to encourage animals to space visits across the day to capture the diurnal variation in enteric CH_4_ production. The pelleted bait feed contained 75% alfalfa meal, 22.85% soybean hulls, 2% liquid molasses, and 0.15% Herd Request (Cares Solutions Co-op, White Cloud, MI) with an average weight of 35 g. Herd Request (Prinova Flavors, LLC, Carol Stream, IL) is a flavoring agent used to encourage animals to visit the AHCS. Pellets were sampled monthly each year and analyzed for nutritive value by a commercial laboratory (DairyLand Laboratories Inc., Arcadia, WI; Table 1). Visits for each individual animal that met the criteria of a good visit were averaged together across the 2-wk sample period to determine emissions. The AHCS was calibrated weekly and monthly CO_2_ recoveries were completed with results falling within 100 ± 5%.

**Table 1.** GreenFeed supplement pellet nutritive value by year

Year	CP, g kg^−1^ DM^1^	aNDF, g kg^−1^ DM	ADF, g kg^−1^ DM	NE_m_, Mcal kg^−1^	NE_g_, Mcal kg^−1^	TDN
2018	181	459	353	57.74	31.87	61.44
2019	175	501	372	55.53	29.85	59.94
2020	184	519	420	54.99	29.36	56.16

^1^CP, crude protein, aNDF, neutral detergent fiber; ADF, acid detergent fiber; NE_m_, net energy for maintenance; NE_g_, net energy for gain; TDN, total digestible nutrients; SIMP, simple forage mixture; COMP, complex forage mixture.

### Forage intake measurements

Forage intake was determined using the dual-marker method described by Karchner (1980) using one experimental unit from each treatment (*n* = 16). The external marker was titanium dioxide (TiO_2_) and the internal marker was indigestible ADF (**iADF**). In 2018, intake was estimated during the last fortnight of the grazing season (September 14 to September 27, 2018). For 2019 and 2020, forage intake was estimated for a fortnight at the beginning and end of each grazing season (May 27 to June 9, 2019 and September 5 to September 18, 2019; June 1 to June 14, 2020 and August 31 to September 13, 2020, respectively). For the first 9 d, animals were bolused daily with 10 g TiO_2_ (Highwater Clays, Ashville, NC) at 0900 hours, and during the last 5 d, animals were bolused and fecal samples were collected via rectal grab at 0900 and 1500 hours. During the fecal collection period, a grazed forage sample was collected from each sampled pasture using the hand-plucking method by a trained observer to mimic the forage selected by the animals grazing in each paddock on day 3 (Gregorini et al., 2006) during the morning grazing bout (0700 to 1000 hours). Diet samples were dried at 60 °C for 48 h to determine DM, then ground through a Wiley mill (Thomas A. Wiley Laboratory Mill, Model 4, Swedesboro, NJ) to pass through a 1-mm screen and transported to East Lansing, MI for analysis.

Fecal samples were immediately placed in a forced-air oven and dried at 60 °C for a minimum 3 d and samples were then checked daily until dried to a constant weight. Dried samples were then ground to pass through a 1-mm screen, then composited across day within animal with a target of 3 g per sample. A subsample of forage and fecal samples were then analyzed for iADF using the procedure described by Bohnert et al. (2002). In duplicate, 0.5 g of each forage and fecal sample were weighed into filter bags (F57; Ankom Technology, Macedon, NY). Forage samples were incubated at 39 °C for 16 h in a solution containing 0.1% pepsin (Catalog #9001-75-6, Fisher Scientific, Hampton, NH) and 10% 1 N HCl using a Daisy^II^ incubator (2 L per incubation vessel; Ankom Technology). Samples were then rinsed with warm (39 °C) tap water and placed in a mesh bag with the fecal samples. All samples were then placed into the rumen of a cannulated dairy cow located at the Michigan State University Dairy Teaching and Research Center (East Lansing, MI) for 96 h. Upon removal, samples were rinsed with warm (39 °C) tap water until the rinse became clear and dried at 50 °C. Samples were then analyzed for iADF content to determine digestibility. Titanium content was determined using mass spectroscopy in triplicate using a modified protocol described by Myers et al. (2004). Composite diet samples were analyzed for crude protein (**CP**), neutral detergent fiber (**aNDF**), acid detergent fiber (**ADF**), net energy for maintenance (**NE**_**m**_), net energy for gain (**NE**_**g**_), and total digestible nutrients via a commercial laboratory (DairyLand Labs Inc., Arcadia, WI) and are presented in Table 2.

**Table 2.** Forage nutritive value of composite diet sample each sampling period estimated via hand plucking

	2018^2^		2019				2020			
	Fall		Summer		Fall		Summer		Fall	
Nutrient, % DM^1^	SIMP	COMP	SIMP	COMP	SIMP	COMP	SIMP	COMP	SIMP	COMP
CP, g kg^−1^ DM	282	282	200	276	193	247	166	168	242	248
aNDF, g kg^−1^ DM	3160	258	416	298	557	452	551	515	487	422
ADF, g kg^−1^ DM	192	176	265	210	346	288	338	317	242	276
NE_m_, Mcal kg^−1^	1.5	1.6	1.3	1.5	1.2	1.3	1.2	1.2	1.3	1.4
NE_g_, Mcal kg^−1^	0.9	1.0	0.8	0.9	0.6	0.8	0.6	0.6	0.7	0.8
TDN	73.9	75.2	68.2	72.5	62.0	66.5	62.6	64.2	63.2	67.4
In situ digestibility, g kg^−1^ DM	79.0	82.2	76.3	86.7	66.1	79.4	71.2	79.2	74.9	68.4

^1^CP, crude protein; aNDF, neutral detergent fiber content; ADF, acid detergent fiber; NE_m_, net energy for maintenance; NE_g_, net energy for gain; TDN, total digestible nutrients; SIMP, simple forage mixture; COMP, complex forage mixture.

^2^Fall 2018 bolusing period, September 14 through September 18; 2019 bolusing periods, May 27 through June 9 (Summer) and September 5 through September 18 (Fall); 2020 bolusing periods, June 1 through June 14 (Summer) and August 31 through September 13 (Fall).

### Forage

Forage samples were taken every 2 wk for the duration of each grazing season. In 2018, estimated forage yield was originally conducted using a rising plate meter (Jenquip, Fielding, New Zealand) with calibrated equations based on clip samples but that method was replaced by the quadrat method after two sampling periods due to difficulty in obtaining representative plate readings for bolting chicory. Each sampling week, before rotating animals pregraze samples were collected by randomly placing four 0.25-m^2^ quadrats and clipping to a 5-cm stubble height in each experimental paddock. Samples were then dried at 60 °C for 48 h to determine DM and forage productivity. The same paddock was then resampled after animals were rotated out using the same sampling procedure to determine DM and postgraze forage residual. Samples were then ground to pass through a 2-mm screen (Wiley Mill) and composited by size for pasture and sample type (pre- or postgrazed) for each week into a 20-g composite sample and transported to Michigan State University Agronomy Farm (East Lansing, MI) where samples were ground to pass through a 1-mm screen (Udy cyclone mill; Model 3010-030, Udy Corporation, Fort Collins, CO). Forage nutritive value was determined using near-infrared reflectance spectroscopy (**NIRS**) for CP, aNDF, ADF, in vitro true dry matter digestibility over 48 h (**IVTDMD48**), lignin, and ash using the grass hay and mixed hay equations for the COMP and SIMP treatments, respectively, sourced from the NIRS Forage and Feed Consortium (Hillsboro, WI). All samples had scanned spectra with a global *H* value less than 3.0 and neighborhood *H* values less than 1.5, indicating that the scanned samples were similar to those of the developed equations. To further validate equations, a subset of 18 samples per treatment across all years were selected to represent the range of scanned samples and sent to a commercial laboratory for chemical analysis (DairyLand Laboratories Inc., Arcadia, WI). For the SIMP treatment, the NIRS equations had a validation *R*^2^ > 0.95 and standard error of prediction less than or equal to 1.6. For the COMP treatment, the NIRS equation had a validation *R*^2^ ≥ 0.86 and standard error of prediction less than or equal to 2.2.

Botanical composition in each experimental unit was determined monthly in each grazing season using the dry-rank-weight method described by Mannetje and Haydock (1963) by two trained observers. In each experimental unit, 24 locations were randomly sampled by placing a 0.13 m^2^ quadrat and ranking species by observed DM content as: 1 (70% of DM), 2 (21% of DM), or 3 (9% of DM). Observers rotated experimental units each sampling period to minimize observer bias.

### Statistical analysis

All data were analyzed using the MIXED procedure in SAS (SAS Institute Inc., Cary, NC, v 9.4). Pasture was considered the experimental unit and included as a random term. Enteric CH_4_, emission intensity (g CH_4_ kg^−1^ gain; **EI**), and CO_2_ were analyzed using the fixed effect of treatment, year, the interaction between treatment and year, and the fixed effect of sex. Visits less than 3 min were removed, animals with less than 10 visits were removed and as were outliers greater than 3 SD from the mean (Alemu et al. 2021). Pasture was considered a random effect along with pasture by year, individual animal nested with year and pasture, and time of sampling nested within year. Emission intensity was calculated using the two shrunk body weights nearest the sampling period to minimize impacts of gains associated with differing points of the grazing season. Forage characteristics were analyzed in a completely randomized design using the MIXED procedure in SAS. Pasture nested in treatment and year by pasture interaction were included as random terms and week was included as a repeated measure. Fixed effects were the treatment, year and the year by treatment interaction. Means were separated using the LSMEANS statement with a Tukey adjustment. Forage intake was analyzed by year due to unequal sampling periods between years. Statistical significance was declared at *P* ≤ 0.05 and tendencies at 0.05 < *P* ≤ 0.10.

## Results

### Forage nutritive value, quantity, and botanical composition

Pre- and postgraze forage mass were significantly impacted by year (*P* < 0.05; Table 3) but neither was impacted by treatment or treatment × year interaction (*P* ≥ 0.48). Forage mass in 2019 tended to be greater than 2018 (*P* = 0.06; 2.92 ± 0.14 vs. 2.40 ± 0.14 t dry matter (**DM**) ha^−1^, respectively). In 2020, pregrazed forage mass tended to be greater than in 2018 (*P* = 0.10). There was no difference in forage mass between 2020 and 2019. Differences observed between postgrazed forage mass were similar to pregrazed forage mass with a significant impact by year (*P* < 0.01). Postgrazed forage mass was lower in 2018, 1.53 ± 0.12 t DM ha^−1^, compared with 2019 and 2020, 2.07 ± 0.11 t DM ha^−1^ and 2.11 ± 0.12 t DM ha^−1^, respectively (*P* < 0.01), but no differences were observed for 2019 and 2020. Within year, there were no observed treatment differences in postgraze forage mass (*P* > 0.10).

**Table 3.** Yearly pre- and postgraze forage mass, t DM ha^−1^

Pregraze forage mass, t DM ha^−1^^1^^,^^2^						
	SIMP	COMP	Average	SEM	*P*-values	
2018	2.45	2.35	2.40*	0.14	Treatment	0.69
2019	2.89	2.95	2.92	0.14	Year	0.05
2020	2.76	3.00	2.80	0.15	Treatment × year	0.71
Postgraze forage mass, t DM ha^−1^						
	SIMP	COMP	Average	SEM	*P*-values	
2018	1.49	1.59	1.53^b^	0.12	Treatment	0.48
2019	1.97	2.18	2.07^a^	0.11	Year	<0.01
2020	2.10	2.12	2.11^a^	0.12	Treatment × year	0.84

^1^SEM, yearly standard error of the mean; SIMP, simple forage mixture; COMP, complex forage mixture.

^2^Differing lowercase superscript within row signifies a significant difference (*P* ≤ 0.05).

*2018 forage mass tended to be lower than 2019 (*P* = 0.06) and 2020 (*P* = 0.10).

**Figure 3.**
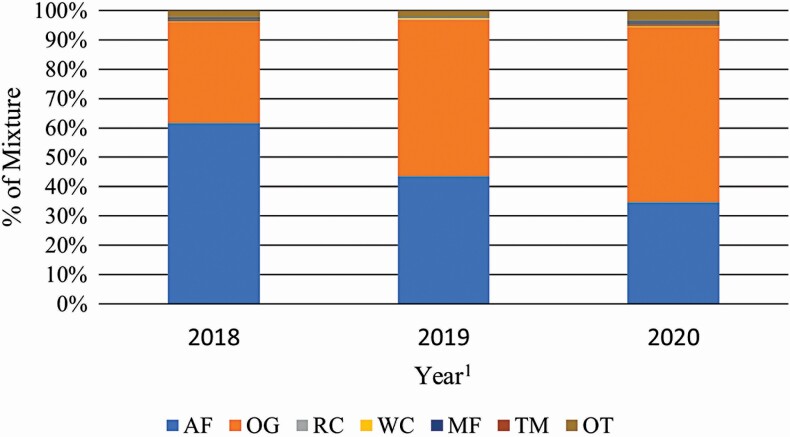
Simple forage mixture botanical composition by year, % DM. ^1^AF, Alfalfa; OG, Orchardgrass; RC, Red Clover; WC, White Clover; MF, Meadow Fescue; Tim, Timothy; OT, Other.

**Figure 4.**
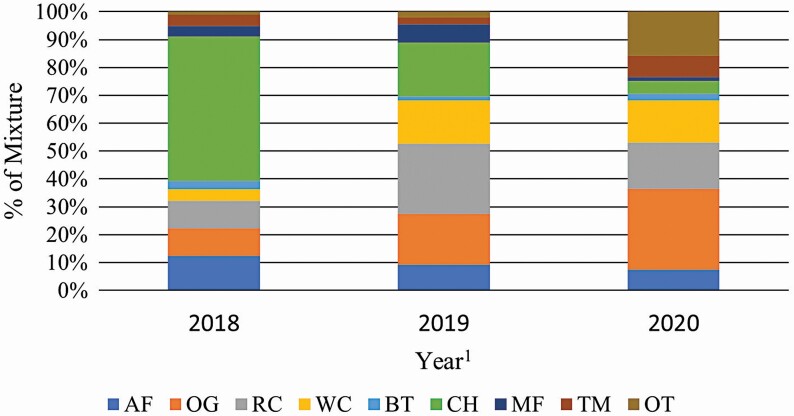
Complex forage mixture botanical composition by year, % DM. ^1^AF, Alfalfa; OG, Orchardgrass; RC, Red Clover; WC, White Clover; BT, Birdsfoot Trefoil; CH, Chicory; MF, Meadow Fescue; Tim, Timothy; OT, Other.

Botanical composition for each treatment is detailed in Figures 3 and 4. Both treatments had considerable annual variation as expected. In 2018, the SIMP forage mixture was dominated by alfalfa at 61.8 ± 2.9% and most of the remaining being 34.7 ± 3.6% orchardgrass. However, by the following year the percentage of alfalfa was lower at 43.8 ± 2.9% (*P* < 0.01) and tended to drop again in 2020 to 34.9 ± 3.2% (*P* = 0.06). This corresponded with yearly increases in the orchardgrass component to 53.7 ± 3.6% in 2019 and 60.0 ± 3.9% in 2020, both greater than in 2018 (*P* < 0.01). For COMP, forage chicory was the dominate species in 2018 at 51.7 ± 2.4% and alfalfa was the second most present species at 12.3 ± 1.5%. All combined, orchardgrass, meadow fescue, and timothy combined for 17.3% of the mixture. In 2019, red clover represented 25.2 ± 1.8% and white clover was 15.5 ± 1.5% of COMP pastures. Forage chicory dropped to 19.4 ± 2.4% (*P* < 0.01) and grass species accounted for 28.4% of the mixture. Similarly, the following year was predominantly a clover-grass mixture with red and white clover accounting for 16.5 ± 2.0% and 15.2 ± 1.7%, respectively, and the grass species accounting for 38.31% of pasture composition. Chicory decreased to 4.6 ± 2.7% (*P* < 0.01) in 2020. Alfalfa concentration stayed relatively consistent from 2018 to 2019, being 12.3 ± 1.5% and 9.1 ± 1.5%, respectively, but lower in 2020 at 7.5 ± 1.6% compared with 2018 (*P* < 0.01).

Pregraze forage nutritive value results for each year is presented in Table 4. There was a year by treatment interaction present for each nutrient (*P* ≤ 0.05), except ADF, and a tendency for an interaction for aNDF content (*P* = 0.07). CP was 22 g kg^−1^ DM less in 2018 for COMP compared with SIMP (*P* < 0.05). There was no observed treatment difference in CP for 2019 or in 2020. CP was less in 2020 compared with previous years (*P* < 0.01) for both treatments.

**Table 4.** Yearly pregraze forage nutritive value estimated via near-infrared reflectance spectroscopy, g kg^−1^ DM

CP^1^	SIMP^2^	COMP	Average	*P*-values	
2018	204^aA^	182^bA^	193	Treatment	0.04
2019	191^aA^	192^aA^	191	Year	<0.01
2020	154^aB^	159^aB^	157	Treatment × year	0.04
Average	185	176			
aNDF	SIMP	COMP	Average		
2018	449^aC^	424^aC^	437	Treatment	0.06
2019	511^aB^	480^aB^	496	Year	<0.01
2020	556^aA^	558^aA^	557	Treatment × year	0.07
Average	488	505			
ADF^3^	SIMP	COMP	Average		
2018			326^B^	Treatment	0.02
2019			343^A^	Year	<0.01
2020			353^A^	Treatment × year	0.82
Average	347^a^	334^b^			
IVTDMD48	SIMP	COMP	Average		
2018	753^bA^	831^aA^	79.22	Treatment	<0.01
2019	775^bA^	808^aA^	79.14	Year	<0.01
2020	764^aA^	773^aB^	76.84	Treatment × year	<0.01
Average	764	804			
Ash	SIMP	COMP	Average		
2018	80^aA^	85^aA^	83	Treatment	0.14
2019	79^aA^	73^aB^	76	Year	<0.01
2020	67^aB^	61^aC^	64	Treatment × year	<0.01
Average	76	73			
Lignin	SIMP	COMP	Average		
2018	68^aA^	73^aA^	70	Treatment	<0.01
2019	56^bB^	71^aA^	63	Year	<0.01
2020	54^aB^	59^aB^	5.67	Treatment × year	<0.01
Average	59	68			

^1^CP, crude protein; aNDF, neutral detergent fiber; ADF, acid detergent fiber; IVTDMD48, in vitro true dry matter digestibility over 48 h.

^2^Differing lowercase superscript within column signifies a significant difference; differing uppercase superscript within row signifies a significant difference (*P* < 0.05); SIMP, simple forage mixture; COMP, complex forage mixture.

^3^ADF is reported as year and treatment means because of the insignificant interaction.

Neutral detergent fiber content was impacted by year (*P* < 0.01) and had a tendency for a treatment × year interaction (*P* = 0.07). Each year aNDF content of each treatment increased (*P* < 0.05). Upon mean separation, the COMP treatment did not differ from SIMP in aNDF concentrations across all years. ADF content ranged from 326 g kg^−1^ DM to 353 g kg^−1^ DM across years and was impacted by treatment and year (*P* ≤ 0.02) but there was no treatment × year interaction (*P* = 0.82). From 2018 to 2019, average ADF content of the treatments increased from 2018 to 2019 by 17 g kg^−1^ DM (*P* < 0.01), but no change was observed from 2019 to 2020. Over the 3 yr, COMP had 13 g kg^−1^ DM less ADF content compared to SIMP. There was a treatment × year interaction for IVTDMD48 (*P* < 0.01). The SIMP treatment had lower IVTDMD48 in 2018 (*P* < 0.01) compared with the COMP, and in 2019 (*P* < 0.01). In the final year treatments did not differ (*P* > 0.10).

Ash content was also impacted by a treatment by year interaction (*P* < 0.01). The SIMP treatment did not differ in ash content in 2018 compared with 2019 but was lower in 2020 (*P* < 0.05). The COMP treatment was lower ash content each year of the study (*P* < 0.05). Lignin content had a treatment by year interaction (*P* < 0.01). In 2018, the treatments did not differ in lignin content. The COMP treatment had similar lignin content in 2019 compared with 2018 and was higher than SIMP that year (*P* < 0.05). The SIMP treatment had less lignin content in 2019 than 2018 (*P* < 0.05) but similar levels in 2020. The COMP treatment had less lignin content in 2020 than previous years (*P* < 0.05). Treatments had similar content of lignin in 2020.

### Forage intake

Forage intake ranged from 5.7 kg DM/animal d^−1^ to 10.9 kg DM d^−1^ over the three grazing seasons. Due to differences in sampling procedure among years, forage intake results were analyzed by year. In 2018, there was no impact of treatment on forage intake during the sampling period (*P* = 0.76; Table 5). In 2019, there was no treatment by sampling period interaction (*P* = 0.28), but there was a period and treatment effect (*P* < 0.01). Cattle in the SIMP treatment consumed less DM (6.8 ± 0.5 kg DM/animal d^−1^) compared with those in COMP (9.0 ± 0.5 kg DM/animal d^−1^). DM intake was greater during the summer sampling period (8.9 ± 0.4 kg DM/animal d^−1^) compared with the fall sampling period (6.9 ± 0.4 kg DM/animal d^−1^). In 2020, there was a treatment by sampling period interaction (*P* < 0.01). In the summer sampling period, cattle grazing the COMP treatment consumed more DM compared with those in SIMP, at 9.7 ± 0.7 kg DM/animal d^−1^ vs. 6.0 ± 0.7 kg DM/animal d^−1^, respectively (*P* = 0.01). The cattle on the COMP treatment had a decrease in DMI between the summer and fall sampling periods, with animals consuming 5.7 ± 0.7 kg DM/animal d^−1^ in the fall period (*P* < 0.01). Intake during the fall sampling period was not different between treatment (7.2 ± 0.7 kg DM/animal d^−1^; *P* = 0.52).

**Table 5.** Forage dry matter intake estimated using the dual-marker method, kg DM/animal d^−1^

2018^1^^,^^2^^,^^3^					
			*P*-values		
	SIMP	COMP	Treatment	Period	Treatment × period
DMI^4^	10.9	10.6	0.76	—	—
SEM	0.8				
2019					
			*P*-values		
	SIMP	COMP	Treatment	Period	Treatment × period
DMI	6.8^b^	9.0^a^	0.01	<0.01	0.28
SEM	0.5	0.5			
	Summer	Fall			
DMI	8.9^A^	6.9^B^			
SEM	0.4	0.4			
2020					
			*P*-values		
	SIMP	COMP	Treatment	Period	Treatment × period
Summer	6.0^bA^	9.7^aA^	0.14	0.07	<0.01
Fall	7.2^aA^	5.7^aB^			
SEM	0.7				

^1^SEM, standard error of the mean; SIMP, simple forage mixture; COMP, complex forage mixture.

^2^Lower case superscript signifies a significant difference between treatment, uppercase superscript signifies a significant difference between period (*P* ≤ 0.05).

^3^2018 sampling periods: September 14 through September 27; 2019 sampling periods: Summer-May 27 through June 9, Fall-September 5 through September 18; 2020 sampling periods: Summer-June 1 through June 14, Fall-August 31 through September 13.

^4^DMI, kg DM forage consumed/animal d^−1^.

Gross energy intake ranged from 114.8 MJ d^−1^ to 215.5 MJ d^−1^ over the 3 yr and results mirrored those of DMI (Table 6). In 2018, animals from the COMP and SIMP treatments consumed similar levels of GE (*P* = 0.55). In 2019, there was no treatment by sampling period interaction (*P* = 0.27), but there were observed treatment and sampling period effects (*P* < 0.01). Animals grazing the SIMP treatment consumed less GE compared with animals grazing the COMP. Again, GEI was significantly less in the fall sampling period for both treatments compared with the summer sampling period. In 2020, there was a treatment by sampling period interaction (*P* = 0.05). In the summer sampling period, animals grazing COMP consumed more GE than those on SIMP (*P* = 0.03). In the fall sampling period, cattle in both treatments consumed similar amounts of GE.

**Table 6.** Gross energy and aNDF intake, MJ/animal d^−1^

GEI^1^^,^^2^^,^^3^						NDFI					
2018			*P*-values			2018			*P*-values		
	SIMP	COMP	Treatment	Period	Treatment × period		SIMP	COMP	Treatment	Period	Treatment × period
GEI	215.5	201.3	0.55	—	—	NDFI	3.5	2.7	0.06	—	—
SEM	16.4					SEM	0.3				
2019			*P*-values			2019					
	SIMP	COMP	Treatment	Period	Treatment × Period		SIMP	COMP	*P*-values		
GEI	129.1^b^	173.1^a^	<0.01	<0.01	0.27	NDFI	3.3	3.3	Treatment	Period	Treatment × Period
SEM	9.9	10.4				SEM	0.2	0.3	0.91	0.13	0.61
Period											
	Summer	Fall									
GEI	169.9^A^	132.3^B^									
SEM	8.4	8.2									
2020			*P*-values			2020			*P*-values		
	SIMP	COMP	Treatment	Period	Treatment × period	NDFI	3.3^bA^	5.0^Aa^	Treatment	Period	Treatment × period
Summer	114.8^bA^	180.3^aA^	0.04	0.58	0.05	SEM	3.5^aA^	2.4^aB^	0.39	<0.01	<0.01
Fall	138.5^aA^	140.3^aA^									
SEM	14.8										

^1^SEM, standard error of the mean; SIMP, simple forage mixture; COMP, complex forage mixture; GEI, gross energy intake from forage in MJ/animal d^−1^; NDFI, neutral detergent fiber consumed from forage kg DM/animal d^−1^.

^2^Lower case superscript signifies a significant difference between treatment, uppercase superscript signifies a significant difference between period (*P* ≤ 0.05).

^3^2018 sampling periods: September 14 through September 27; 2019 sampling periods: Summer-May 27 through June 9, Fall-September 5 through September 18; 2020 sampling periods: Summer-June 1 through June 14, Fall-August 31 through September 13.

aNDF intake tended to be different between the treatments in 2018 (*P* = 0.06; Table 6). In 2019, there was no treatment by time interaction, treatment effect, or effect of sampling period (*P* ≥ 0.13). In 2020, there was a significant treatment by sampling period interaction (*P* < 0.01). During the summer sampling period, animals on the SIMP treatment consumed less NDF (*P* = 0.02) compared with those on the COMP. The animals grazing the COMP treatment had a significant (*P* < 0.01) reduction in NDFI between summer and fall sampling. The SIMP and COMP were not different between fall sampling period that year.

### Animal performance and emissions

Animal performance results are displayed in Table 7. Animal liveweight gain had a treatment by year interaction (*P* = 0.04). However, upon mean separation no difference between treatments within years was observed. In 2018, live weight gain (**LWG**) was similar between treatments, 96.77 ± 2.10 vs. 101.79 ± 2.11 kg LWG for SIMP and COMP, respectively, and was similar to the results observed in 2020, 104.40 ± 1.93 vs. 98.85 ± 1.92 kg LWG for SIMP and COMP, respectively. Performance during 2018 and 2020 production years was less than the LWG observed in 2019 where the SIMP treatment had an LWG of 128.90 ± 1.92 kg and the COMP had an LWG of 128.79 ± 1.92 kg. There was a sex effect on LWG with steers gaining more weight than heifers (*P* < 0.01; 120.74 ± 1.62 vs. 99.10 ± 1.30 kg LWG, respectively). Similarly, when considered on a daily basis, animal performance was impacted by year (*P* < 0.01; Table 7). In 2020, animals gained significantly less than prior years at 0.84 ± 0.03 kg d^−1^ compared to 1.02 ± 0.03 and 0.96 ± 0.03 kg d^−1^ for 2018 and 2019, respectively. The improved LWG in 2019 was a result of increased grazing duration compared with other years but was not due to improved animal performance compared to the 2018 grazing season. There was a significant impact of sex on ADG (*P* = 0.04) with heifers gaining 0.90 ± 0.02 kg d^−1^ compared with 0.98 ± 0.03 kg d^−1^ for steers.

**Table 7.** Animal performance and greenhouse gas emissions while grazing treatment pastures

	Live weight gain (kg)^1^^,^^2^			*P*-value			
Year	SIMP	COMP	SEM	Treatment	Year	Treatment × year	Sex
2018	96.77^aB^	101.79^aB^	1.73	0.89	<0.01	0.04	<0.01
2019	128.90^aA^	128.79^aA^	1.50				
2020	104.40^aB^	98.85^aB^	1.50				
Sex	LWG (kg)		SEM				
Heifer	99.10^b^		1.30				
Steer	120.74^a^		1.62				
	Average daily gain			*P*-value			
Year	ADG (kg)^3^		SEM	Treatment	Year	Treatment × year	Sex
2018	1.02^A^		0.03	0.81	<0.01	0.44	0.04
2019	0.96^A^		0.03				
2020	0.84^B^		0.03				
Sex	ADG (kg)		SEM				
Heifer	0.90^b^		0.02				
Steer	0.99^a^		0.03				
	CH_4_ g d^−1^		SEM	*P*-value			
SIMP	211.1		5.4	Treatment	Year	Treatment × year	Sex
COMP	193.4*		5.5	0.06	0.44	0.33	0.90
	CO_2_ g d^−1^		SEM	*P*-value			
SIMP	7,063.5		169.2	Treatment	Year	Treatment × year	Sex
COMP	6,934.8		169.1	0.60	0.43	0.64	0.46
	Emission intensity (g CH_4_ kg^−1^ gain)		SEM	*P*-value			
SIMP	240.9		15.3	Treatment	Year	Treatment × year	Sex
COMP	227.3		16.1	0.56	0.36	0.52	0.81

^1^SEM, standard error of the mean; SIMP, simple forage mixture; COMP, complex forage mixture.

^2^Differing lowercase superscripts signify a significant difference between treatments within year, differing uppercase letters signify a significant difference between year respective of treatment (*P* ≤ 0.05).

^3^ADG, average daily gain in kg d^−1^.

*COMP tended to have lower CH_4_ g d^−1^ compared with SIMP (*P* = 0.06).

Enteric methane emissions were not impacted by the treatment by year interaction (*P* = 0.33) but tended to be impacted by treatment (*P* = 0.06; Table 7). Neither year nor sex impacted enteric CH_4_ emission rate (*P* ≥ 0.44). Cattle grazing COMP had lower enteric CH_4_, 193.4 ± 5.5 g CH_4_ d^−1^, than SIMP, 211.1 ± 5.4. Emission intensity (Table 7) was not impacted by any variable (*P* ≥ 0.36). Similarly, CO_2_ emissions were not affected by treatment, year, their interaction, or sex (*P* ≥ 0.43).

## Discussion

The hypothesis that the COMP forage mixture would result in greater forage productivity was rejected in this experiment. Forage productivity was similar between treatments across all 3 yr and previous literature is inconsistent on the differences in forage productivity with increasing levels of forage diversity (Sanderson et al., 2007; Deak et al., 2007). Small plot studies have indicated that there is no benefit to planting forage mixtures containing more than four species (Tracy and Sanderson, 2004). However, in a grazing experiment examining forage mixtures with increasing levels of species inclusion, Deak et al. (2004) found that greater yields were associated with the inclusion of red clover. Similarly, Sanderson et al. (2016) observed a positive relationship between the number of species planted and annual DM yield and that red clover contributed significantly to DM productivity in the first 2 yr of the study. High concentrations of forage chicory have also been shown to increase forage biomass (Sanderson et al., 2007), but that was not observed in this study when forage chicory accounted for 52% of pasture DM in the first year. In 2018, forage quantity was higher for the COMP treatment early in the grazing season as forage chicory encountered favorable environmental conditions (data not shown); however, bolted chicory pastures were mechanically topped by clipping to a 25-cm stubble height (Li and Kemp, 2005). Rainfall in June of 2018 was lower than other years of the study and the mechanical topping that occurred that month may explain why chicory did not increase pasture productivity that year. In subsequent years early season forage growth was managed more aggressively in the COMP mixture by rotating cattle daily to reduce the likelihood of chicory bolting. Some research has suggested that increased forage diversity may result in improved forage productivity during dry years (Sanderson et al., 2005, 2007; Isbell, 2015). However, Deak et al. (2007) observed that complexity of forage mixtures does not always translate to improve forage productivity and that individual species in the mixtures is more important. The results of this study would indicate that no advantage is gained through the adoption of either forage mixture, even with shifting botanical composition.

The shift in botanical composition of both treatments toward greater grass species was expected (Tracy et al., 2018). These results are similar to those reported by Sanderson et al. (2005) in the Northeastern United States. The authors examined different forage mixtures containing two, three, six, and nine species of grass, forbs, and legumes grazed by lactating dairy cows. They reported that, after 2 yr of grazing, orchardgrass dominated the pastures at the start of the third year after stand establishment, similar to the results of both mixtures utilized in this trial. Additionally, they found that chicory and legumes would have to be reestablished frequently to remain in the mixtures. Similarly, in a review of plot- and pasture-scale experiments, Sanderson et al. (2007) reported that nearly one-half of species in complex mixtures did not persist past the third or fourth year. The short-lived contribution of chicory to the botanical composition of the complex pastures agrees with previous literature (Belesky et al., 1999; Sanderson et al., 2003, 2005; Labreveux et al., 2004). Under grazing, chicory persistence has shown to decrease over time, but can withstand heavier defoliation under rotational grazing as new shoots regenerate from the basal crown (Rumball, 1986). However, spring and autumn grazing management is critical for its persistence over time (Li and Kemp, 2005) and the increased defoliation at the start of the grazing season in 2019 and 2020 may partially explain its lack of persistence. The combination of mechanical topping in 2018 and frequent grazing events may explain the prevalence of red and white clover in the COMP pastures in 2019 and 2020. Frequent defoliation of forages keeps grass species short, therefore decreasing the shading effect on forage species lower in canopy (Wong and Wilson, 1980; Groya and Sheaffer, 1981; Chiavegato et al., 2015). This perhaps allowed these species to remain competitive as the COMP treatment shifted to grass-dominated pastures in 2020. The reduction in red clover from 2019 to 2020 would agree with previous studies that white clover is more long-lived in forage mixtures (Sanderson et al., 2016). The decline in alfalfa concentration was expected for the SIMP forage treatment. In alfalfa-grass mixtures, grasses tend to dominate the mixture over time (Berdahl et al., 2004; Baxter et al., 2017). Alfalfa stands self-thin over time and are susceptible to winter-kill, although grass mixtures can provide some protection (Malhi et al., 2002), and therefore provides grass species more space to proliferate (Aponte et al., 2019).

The forage nutritive values reported here are within the range of reported values for cool-season forages in the region (Muller and Fales, 1998; Cassida et al., 2000; Soder et al., 2006; Sanderson et al., 2016). Shifts in forage nutritive value mirrored those of botanical composition with treatment paddocks being similar in forage nutritive value in 2020 when both treatments were dominated by grass species. The hypothesis that the COMP forage treatment would have improved forage nutritive value compared with the SIMP treatment would agree with the IVTDMD48 and ADF values observed in this experiment. Although birdsfoot trefoil did not establish as desired, the high forage chicory concentration in 2018 and clover content in following years may explain the difference observed here (Labreveux et al., 2004; Soder et al., 2006; Mangwe et al., 2020). Additionally, both grazing treatments were dominated by grass species in 2020 could explain the convergence between the two treatments in nutritive value that year, particularly in IVTDMD48 in COMP pastures (Deak et al., 2007; Sanderson et al., 2016). Sanderson et al. (2016) reported that digestibility was negatively related to grass percentage and aNDF content was positively related to grass content. Similarly, Deak et al. (2007) reported changes in nutritive value in complex mixtures over multiple years were explained by proportion of grasses to legumes.

We anticipated that grazing the COMP treatment would result in reduced enteric CH_4_ emissions compared to those grazing the SIMP treatment. The results of this experiment reject this hypothesis, however there was a tendency for the COMP to have lower enteric CH_4_ on a g d^−1^ basis. This may partially be explained by the relatively high nutritive value of both treatments. As shown in Table 2 from hand-plucked diet samples during forage intake periods, animals potentially selected high-quality diets even during periods of low forage nutritive value. Few studies have considered how species diversity impacts animal performance, as most studies examine mono- or two-species mixtures (Soder et al., 2007; Alemu et al., 2019). However, these results are similar to those reported by Soder et al. (2006) on grazing dairy cows. They tested four forage mixtures containing an increasing number of species and observed that dairy cows grazing complex mixtures had no decreased performance compared with those of simple mixtures. In a comparison of steers grazing 7- and 12-seed mixtures, Alemu et al. (2019) reported lower ADG values for rotationally grazed steers (0.80 kg d^−1^) and no difference in animal performance between the different mixtures but did between years, similar to this study. Tracy and Faulkner (2006) did a study comparing grazing beef cow–calf pairs on pastures containing 3-, 5-, or 8-forage species over 3 yr and reported no impact of species richness on animal performance.

Enteric CH_4_ emissions reported in this study were within the range reported by others examining grazing beef cattle including DeRamus et al. (2003) and Pavao-Zuckerman et al. (1999). McCaughey et al. (1997) evaluated steers grazing alfalfa/grass pasture and found enteric CH_4_ ranged from 171 to 217 g d^−1^, in line with the current study. However, in a similar study comparing yearling beef heifers at similar weights, grazing alfalfa-meadow bromegrass (*Bromus biebersteinii*; 40% alfalfa, 60% meadow bromegrass) vs. 100% meadow bromegrass pastures at similar nutritive values to the current experiment, enteric CH_4_ emissions were lower than those reported here (142.8 to 167.6 g CH_4_ d^−1^ for cattle grazing alfalfa-meadow bromegrass; Chaves et al., 2006). A potential explanation for this difference between the two studies is the enteric CH_4_ values reported here are grazing season means rather than a 5-d sampling period. Enteric CH_4_ emissions tended to be lower for COMP, but no difference was observed for EI. The shift in botanical composition we observed and its impacts on rumen function could explain these results. These results are similar to those of Jonker et al. (2018) comparing a ryegrass:white clover mixture, containing both perennial and annual ryegrass species, to a diverse grass, legume and herb mixture. They found no difference in EI between the forage mixtures but there were changes per unit of DM consumed. In this experiment, intake was higher in 2019 and the first sampling period in 2020 for COMP. This could be due to forage diversity, as some research has suggested that increased forage diversity may result in increases in DMI (Wang et al., 2010) and(or) changes in rumen function depending on the forage species present (Jung and Allen, 1995). Additionally, this could indicate that animals grazing SIMP pastures were able to capture similar gains while consuming less forage through more complete fermentation of rumen digestible nutrients. This could have resulted from the grass/legume mixture having reduced rumen passage rate, and therefore more complete digestion of potentially digestible nutrients, which drove enteric CH_4_ to be higher per unit of intake but kept performance the same (Jung and Allen, 1995). The high chicory content in year 1 and high clover content in years 2 and 3 for COMP may have resulted in increased rumen passage rate, reduction in rumen degradation, higher DMI and suppressed methane per unit of intake (Steg et al., 1994; Freudenberger et al., 1994; Navarro-Villa et al., 2011). Navarro-Villa et al. (2011) examined the impact of clover inclusion on in vitro methane emissions and found that red clover had reduced emissions per unit of DM incubated compared with perennial ryegrass, but when expressed per unit of DM digested ryegrass had lower emissions, agreeing with Jonker et al. (2018). In a study examining the impact of multiple different forage species on enteric CH_4_ production in sheep, Waghorn et al. (2002) hypothesized that the beneficial impact of red clover was due to its lower fiber content and faster rate of passage. Additionally, Aitchison et al. (1986) found that clover hay had significantly faster rates of digestion of DM and NDF compared with grass hay, and that rumen pool sizes were lower for sheep offered clover hay. The 50% inclusion of chicory in year 1 may have had similar impacts on rumen fermentation as clover (Waghorn et al., 2002; Hristov et al., 2013; Mangwe et al., 2020). Inclusion of forage chicory has inconsistent results on enteric CH_4_ (Sun et al., 2011; Williams et al., 2016) and most studies have not found it to be a viable option for CH_4_ reduction. Mangwe et al. (2020), in a study examining grazing dairy cows at 50% inclusion level of chicory, observed no changes in DMI compared with those grazing perennial ryegrass, although cows had improved performance, and lower ruminal pH due to increased rumen VFA production. The animals in this experiment grazing COMP in 2018 may have had an altered rumen VFA profile and decreased rumination time which resulted in lower enteric CH_4_. Similarly, Waghorn et al. (2002) found that sheep fed forage chicory produced 30% less enteric CH_4_, similar to red clover in that study, than those offered alfalfa or a perennial ryegrass:white clover mixture. This was hypothesized to be due to the lower fiber content and high digestibility in chicory. One limitation with this experiment was the use of a single GreenFeed unit, which inhibited our ability to sample both treatments simultaneously and to directly compare emissions per unit of intake. Additionally, this sampling structure could be limiting the precision of this experiment when comparing emission rates.

Additionally, while we observed no consistent difference between the two treatments during intake measurement periods, we may not have consistently captured voluntary feed intake from these animals. To test this, we calculated individual animal intake during the sampling periods using equations recommended for yearling cattle by the National Research Council Nutrient Requirements for Beef Cattle (NASEM, 2016). The calculated range of expected intakes was higher than animal measurements at 7.6 to 11.1 kg DMI d^−1^ vs. 5.7 to 10.9 kg DMI d^−1^, respectively. Comparing the treatment, year, period combinations the lowest values from the dual-marker calculation were increased using the NRC calculation, with the reduction in forage intake in 2019 from the summer to fall sampling period removed. Additionally, the low values observed in 2020 and treatment differences in 2019 were not present using the NASEM calculation. Chaves et al. (2006) showed similar results when comparing the alkane method with the Cornell Net Protein Carbohydrate System for predicting intake. These short-term, indirect methods of calculating intake are known to have a high degree of variability due to the use of a marker to estimate fecal output, the reliance on the quality of the collected representative forage sample, and the variance in animal selectivity over a short sampling period (Galyean and Gunter, 2016). Additionally, forage diversity and varying rate of passage could explain these results. This experiment also relied on hand-plucking forage samples which has been shown previously to have operator bias and potentially over-estimate the quality of high-quality forages (Langlands, 1974; De Vries, 1995). In this experiment, the changes in DMI were inconsistent and when DMI was estimated using NASEM (2016) equations there was no difference between the treatments.

## Conclusions

The objective of this experiment was to examine the forage productivity, forage nutritive value, animal performance and enteric CH_4_ emissions of two common Midwest grazing forage mixtures: a simple alfalfa:orchardgrass mixture, and a complex forage mixture. The COMP and SIMP mixtures resulted in similar animal performance, forage productivity, but the COMP mixture had lower ADF content and IVTDMD48 over the first 2 yr of the study. Additionally, animals grazing COMP tended to have lower enteric CH_4_ than SIMP on a g d^−1^ basis, but this did not result in improved EI. This would agree with previous literature showing that grazing pastures with differing forage diversity can result in similar animal performance. Additionally, both mixtures had species with poor persistence (alfalfa and forage chicory) that would need to be reseeded regularly to retain them in the mixture. This experiment also serves to supplement the dearth of literature examining animal emissions when grazing complex forage mixtures and continued research is needed to confirm the results of this experiment and to test other producer relevant mixtures.

## Abbreviations

ADF: acid detergent fiber
ADG: average daily gain
aNDF: neutral detergent fiber
CP: crude protein
DM: dry matter
DMI: dry matter intake
EI: emission intensity
iADF: indigestible acid detergent fiber
LWG: live weight gain
NE_g_: net energy for gain
NE_m_: net energy for maintenance
NIRS: near-infrared reflectance spectroscopy
TDN: total digestible nutrients

## References

[CIT0001] Aitchison, E. M., M. Gill, M. S. Dhanoe, and D. F. Osbourn. 1986. The effects of digestibility and forage species on the removal of digesta from the rumen and the voluntary intake of hay by sheep. Br. J. Nutr. 56:463–476. doi:10.1079/BJN19860126 3676225

[CIT0002] Alemu, A. W., H. Janzen, S. Little, X. Hao, D. J. Thompson, V. Baron, A. Iwaasa, K. A. Beauchemin, and R. Kröbel. 2017. Assessment of grazing management on farm greenhouse gas intensity of beef production systems in the Canadian Prairies using life cycle assessment. Agric. Syst. 158:1–13. doi:10.1016/j.agsy.2017.08.003

[CIT0003] Alemu, A. W., R. Kröbel, B. G. McConkey, and A. D. Iwaasa. 2019. Effect of increasing species diversity and grazing management on pasture productivity, animal performance, and soil carbon sequestration of re-established pasture in Canadian prairie. Animals. 9:127. doi:10.3390/an9040127 PMC6523940 30934844

[CIT0004] Alemu, A. W., A. L. Shreck, C. W. Booker, S. M. McGinn, L. K. D. Pekrul, M. Kindermann, and K. A. Beauchemin. 2021. Use of 3-nitrooxypropanol in a commercial feedlot to decrease enteric methane emissions from cattle fed a corn-based finishing diet. J. Anim. Sci. 99:1–13. doi:10.1093/jas/skaa394 PMC8355502 33515476

[CIT0005] Aponte, A., D. Samarappuli, and M. T. Berti. 2019. Alfalfa-grass mixtures in comparison to grass and alfalfa monocultures. Agron. J. 111:628–638. doi:10.2134/agronj2017.12.0753

[CIT0006] Archimède, H., M. Eugene, C. M. Magdeleine, M. Boval, C. Martin, D. P. Morgavi, P. Lecomte, and M. Doreau. 2011. Comparison of methane production between C3 and C4 grasses and legumes. Anim. Feed Sci. Technol. 166-167:59–64. doi:10.1016/j.anifeedsci.2011.04.003

[CIT0007] Barbehenn, R. V., Z. Chen, D. N. Karowe, and A. Spickard. 2004. C_3_ grasses have higher nutritional quality than C4 grasses under ambient and elevated atmospheric CO_2_. Global Change Biol. 10:1565–1575. doi:10.1111/j.1365-2486.2004.00833.x

[CIT0009] Baxter, L., C. West, P. Brown, and P. Green. 2017. Nondestructive determination of legume content in grass-legume pastures. CFTM. 3:cftm2016.12.008. doi:10.2134/cftm2016.12.008

[CIT0010] Beauchemin, K. A., M. Kreuzer, F. O’Mara, and T. A. McAllister. 2008. Nutritional management for enteric methane abatement: a review. Aust. J. Exp. Agric. 48:21–27. doi:10.1071/EA07199

[CIT0013] Belesky, D. P., J. M. Fedders, K. E. Turner, and J. M. Ruckle. 1999. Productivity, botanical composition, and nutritive value of swards including forage chicory. Agron. J. 91:450–456. doi:10.2134/agronj1999.00021962009100030015x

[CIT0014] Berdahl, J., J. Karn, and J. Hendrickson. 2004. Nutritive quality of cool season grass monocultures and binary grass-alfalfa mixtures at late harvest. Agron. J. 96:951–955. doi:10.2134/agronj2004.0951

[CIT0015] Boadi, D., C. Benchaar, J. Chiquette, and D. Masse. 2004. Mitigation strategies to reduce enteric methane emissions from dairy cows: update review. Can. J. Anim. Sci. 84: 319–335. doi:10.4141/A03-019

[CIT0017] Bohnert, D. W., C. S. Schauer, S. J. Falck, and T. DelCurto. 2002. Influence of rumen protein degradability and supplementation frequency on steers consuming low-quality forage: II. Ruminal fermentation characteristics. J. Anim. Sci. 80:2978–2988. doi:10.2527/2002.80112978x. 12462267

[CIT0018] Cassida, K. A., T. S. Griffin, J. Rodriguez, S. C. Patching, O. B. Hesterman, and S. R. Rust. 2000. Protein degradability and forage quality in maturing alfalfa, red clover and birdsfoot trefoil. Crop Sci. 40-209-215. doi:10.2135/cropsci2000.401209x

[CIT0019] Chaves, A. V., L. C. Thompson, A. D. Iwaasa, S. L. Scott, M. E. Olson, C. Benchaar, D. M. Veira, and T. A. McAllister. 2006. Effect of pasture type (alfalfa vs. grass) on methane and carbon dioxide production by yearling beef heifers. Can. J. Anim. Sci. 86:409–418. doi:10.4141/A05-81

[CIT0020] Chiavegato, M. B., J. E. Rowntree, D. Carmichael, and W. J. Powers. 2015. Enteric methane from lactating beef cows managed with high- and low-input grazing systems. J. Anim. Sci. 93:1365–1375. doi:10.2527/jas.2014-8128. 26020913

[CIT0021] De Vries, M. F. W . 1995. Estimating forage intake and quality in grazing cattle: A reconsideration of the hand-plucking method. J. Range Mangage. 46:370–375.

[CIT0022] Deak, A., M. H. Hall, and M. A. Sanderson. 2004. Forage production and forage mixture complexity. Proc. Am. Forage Grassl. Counc. 13:220–224.

[CIT0023] Deak, A., M. H. Hall, M. A. Sanderson, and D. D. Archibald. 2007. Production and nutritive value of grazed simple and complex forage mixtures. Agron. J. 99:814–821. doi:10.2134/agronj2006.0166

[CIT0024] DeRamus, H. A., T. C. Clement, D. D. Giampola, and P. C. Dickison. 2003. Methane emissions of beef cattle on forages: efficiency of grazing management systems. J. Environ. Qual. 32:269–277. doi:10.2134/jeq2003.2690. 12549566

[CIT0026] FAO. 2016. Environmental performance of large ruminant supply chains: guidelines for assessment Livestock Environmental Assessment and Performance Partnership. Rome (Italy): FAO.

[CIT0027] Freudenberger, D. O., C. J. Burns, K. Toyokawa, and T. N. Barry. 1994. Digestion and rumen metabolism of red clover and perennial ryegrass/white clover forages by red deer. J. Agric. Sci. 122:115–120. doi:10.1017/S0021859600065850

[CIT0028] Galyean, M. L., and S. A. Gunter. 2016. Predicting forage intake in extensive grazing systems. J. Anim. Sci. 94:26–43. doi:10.2527/jas.2016-0523

[CIT0029] Gregorini, P., M. Eirin, R. Regi, M. Ursion, O. E. Ansin, and S. A. Gunter. 2006. Timing of herbage allocation in strip grazing: effects on grazing and performance of beef heifers. J. Anim. Sci. 84:1943–1950. doi:10.2527/jas.2005-537 16775079

[CIT0030] Groya, F. L., and C. C. Sheaffer. 1981. Establishment of sod-seeded alfalfa at various levels of soil moisture and grass competition. Agron. J. 73:560–565. doi:10.2134/agronj198100021962007300030036x

[CIT0032] Hristov, A. N., J. Oh, J. L. Dijkstra, E. Kebreab, G. Waghorn, H. P. S. Makkar, A. T. Adesogan, W. Yang, C. Lee, P. J. Gerber, et al. 2013. Mitigation of methane and nitrous oxide emissions from animal operations: I. a review of enteric methane mitigation options. J. Anim. Sci. 91:5045–5069. doi:10.2527/jas.2013-65834 24045497

[CIT0033] Isbell, F . 2015. Agroecology: agroecosystem diversification. Nat. Plants 1:15041. doi:10.1038/nplants.2015.41. 27247041

[CIT0034] Jonker, A., L. Farrell, D. Scobie, R. Dynes, G. Edwards, H. Hague, R. McAuliffe, A. Taylor, T. Knight, and G. Waghorn. 2018. Methane and carbon dioxide emissions from lactating dairy cows grazing mature ryegrass/whiteclover or a diverse pasture comprising ryegrass, legume and herbs. Anim. Prod. Sci. 59:1063–1069. doi:10.1071/AN18019

[CIT0035] Jung, H. G., and M. S. Allen. 1995. Characteristics of plant cell walls affecting intake and digestibility of forages by ruminants. J. Anim. Sci. 73:2774–2790. doi:10.2527/1995.7392774x. 8582870

[CIT0036] Karchner, R. J . 1980. Effects of protein and energy supplementation of cows grazing native winter range forage on intake and digestibility. J. Anim. Sci. 51:432–438. doi:10.2527/jas1980.512432x

[CIT0037] Labreveux, M., M. H. Hall, and M. A. Sanderson. 2004. Productivity of chicory and plantain cultivars under grazing. Agron. J. 96:710–716. doi:10.2134/agronj2004.0710

[CIT0039] Langlands, J. P . 1974. Studies on the nutritive value of the diet selected by grazing sheep VII. A note on hand plucking as a technique for estimating dietary composition. Anim. Sci. 19:249–252. doi:10.1017/S0003356100022807

[CIT0040] Li, G., and P. D. Kemp. 2005 Forage chicory (*Cichorium intybus* L.): a review of its agronomy and animal production. Adv. Agronomy. 88:187–222. doi:10.1016/S0065-211(05)88005-8

[CIT0041] Malhi, S. S., R. P. Zentner, and K. Heier. 2002. Effectiveness of alfalfa in reducing fertilizer N inputs for optimum forage yield, protein concentration, returns and energy performance of bromegrass-alfalfa mixtures. Nutr. Cycling Agroecosyst. 62:219–227. doi:10.1023/A:1021229824357

[CIT0042] Mangwe, M., R. Bryant, and P. Gregorini. 2020. Rumen fermentation and fatty acid composition of milk of mid lactating dairy cows grazing chicory and ryegrass. Animals. 10:169. doi:10.3390/ani10010169 PMC7023442 31963810

[CIT0043] Mannetje, L. T., and K. P. Haydock. 1963. The dry-weight-rank method for the botanical analysis of pasture. Grass Forage Sci. 18:268–275. doi:10.1111/j.1365-2494.1963.tb00362.x

[CIT0044] McCaughey, W. P., K. Wittenberg, and D. Corrigan. 1997. Methane production by steers on pasture. Can. J. Anim. Sci. 77:519–524. doi:10.4141/A96-137

[CIT0046] Muller, L. D., and S. L. Fales. 1998. Supplementation of cool-season grass pastures for dairy cattle. In: J. H. Cherney and D. J. R. Cherney, editors. Grass for dairy cattle. Oxon, UK: CAB International; p. 335–350.

[CIT0047] Myers, W. D., P. A. Ludden, V. Nayigihugu, and B. W. Hess. 2004. Technical note: a procedure for the preparation and quantitative analysis of samples for titanium dioxide. J. Anim. Sci. 82:179–183. doi:10.2527/2004.821179x. 14753360

[CIT0073] National Academies of Sciences, Engineering, and Medicine (NASEM). 2016. Nutrient requirements of beef cattle: eighth revised edition. Washington, DC: The National Academies Press. doi:10.17226/19014 38386771

[CIT0048] National Oceanic and Atmospheric Administration. 2020. Lake City Experimental Farm Daily Summaries Page. https://www.ncdc.noaa.gov/cdo-web/datasets/GHCND/stations/GHCND:USC00204502/detail (accessed November 17, 2020).

[CIT0049] Navarro-Villa, A., M. O’Brien, S. Lopez, T. M. Boland, and P. O’Kiely. 2011. In vitro rumen methane output of red clover and perennial ryegrass assayed using the gas production technique (GPT). Anim. Feed Sci. and Tech. 168:152–164. doi:10.1016/j.anifeedsci.2011.04.091

[CIT0051] Pavao-Zuckerman, M. A., J. C. Waller, T. Ingle, and H. A. Fribourg. 1999. Methane emissions of beef cattle crazing tall fescue pastures at three levels of endophyte infestation. J. Environ. Qual. 28:1963–1969. doi:10.2134/jeq1999.00472425002800060036x

[CIT0052] Rotz, C. A., S. Asem-Hiablie, S. E. Place, and G. Thoma. 2019. Environmental footprints of beef cattle production in the United States. Agric. Syst. 169:1–13. doi:10.1016/j.agsy.2018/11.005

[CIT0053] Rumball, W . 1986. Grasslands Puna chicory (Cichorium intybus L.). NZ J. Exp. Agric. 14:105–107. doi:10.1080/03015521.1986.10426133

[CIT0054] Sanderson, M. A., S. C. Goslee, K. J. Soder, R. H. Skinner, B. F. Tracy, and A. Deak. 2007. Plant species diversity, ecosystem function, and pasture management: A perspective. Can. J. Plant Sci. 87:479–487. doi:10.4141/P06-135

[CIT0055] Sanderson, M. A., M. Labrevuex, M. H. Hall, and G. F. Elwinger. 2003. Forage yield and persistency of chicory and English plantain. Crop Sci. 43:995–1000. doi:10.2135/cropsci2003.9950

[CIT0056] Sanderson, M. A., R. H. Skinner, D. J. Barker, G. R. Edwards, B. F. Tracy, and D. A. Wedin. 2004. Plant species diversity and management of temperate forage and grazing land ecosystems. Crop Sci. 44:1132–1144. doi:10.2135/cropsci2004.1132

[CIT0057] Sanderson, M. A., K. J. Soder, L. D. Muller, K. D. Klement, R. H. Skinner, and S. C. Goslee. 2005. Forage mixture productivity and botanical composition in pastures grazed by dairy cattle. Agronomy J. 97:1465–1471. doi:10.2134/agronj2005.0032

[CIT0058] Sanderson, M. A., R. Stout, and G. Brink. 2016. Productivity, botanical composition, and nutritive value of commercial pasture mixtures. Agron. J. 108:93–100. doi:10.2134/agronj15.0259

[CIT0059] Soder, K. J., A. J. Rook, M. A. Sanderson, and S. C. Goslee. 2007. Interaction of plant species diversity on grazing behavior and performance of livestock grazing temperate region pastures. Crop Sci. 47:416–425. doi:10.2135/cropsci2006.01.0061

[CIT0060] Soder, K. J., M. A. Sanderson, J. L. Stack, and L. D. Muller. 2006. Intake and performance of lactating cows grazing diverse forage mixtures. J. Dairy Sci. 89:2158–2167. doi:10.3168/jds.S0022-0302(06)72286-X. 16702282

[CIT0062] Stanley, P. L., J. E. Rowntree, D. K. Beede, M. S. DeLonge, and M. W. Hamm. 2018. Impacts of soil carbon sequestration on life cycle greenhouse gas emissions in Midwestern USA beef finishing systems. Agric. Syst. 162:249–258. doi:10.1016/j.agsy.2018.02.003

[CIT0063] Steg, A., W. M. van Straalen, V. A. Hindle, W. A. Wensink, F. M. H. Dooper, and R. L. M. Schils. 1994. Rumen degradation and intestinal digestion of grass and clover at two maturity levels during the season in dairy cows. Grass Forage Sci. 49:378–390. doi:10.111/j.1365-2494.1994.tb02014.x

[CIT0064] Sun, X. Z., S. O. Hoskin, S. Muetze, G. Molano, and H. Clark. 2011. Effects of forage chicory (*Cichorium intybus*) and perennial ryegrass (*Lolium perenne*) on methane emissions in vitro and from sheep. Anim. Feed. Sci. and Tech. 166-167:391–397. doi:10.1016/j.anifeedsci.2011.04.027

[CIT0065] Thompson, L. R., and J. E. Rowntree. 2020. Invited Review: Methane sources, quantification, and mitigation in grazing systems. Appl. Anim. Sci. 36:556–573. doi:10.15232/aas.2019-01951

[CIT0066] Tracy, B. F., and D. B. Faulkner. 2006. Pasture and cattle responses in rotationally stocked grazing systems sown with differing levels of species richness. Crop Sci. 46:2062–2068. doi:10.2135/cropsci2005.12.0473

[CIT0067] Tracy, B. F., and M. Sanderson. 2004. Relationships between forage plant diversity and weed invasion in pasture communities. Agric. Ecosyst. Environ. 102:175–183.

[CIT0074] Tracy, B. F., K. Albrecht, J. Flores, M. Hall, A. Islam, G. Jones, W. Lamp, J. W. MacAdam, H. Skinner, and C. Teutsch. 2018. Evaluating grass-legume forage mixtures across different environments. Crop Soils. 51:30–47. doi:10.2134/cs2018.51.0303

[CIT0068] Velazco, J. I., D. G. Mayer, S. Zimmerman, and R. S. Hegarty. 2016. Use of short-term breath measures to estimate daily methane production by cattle. Animal 10:25–33. doi:10.1017/S1751731115001603. 26303821

[CIT0069] Waghorn, G. C., M. H. Tavendale, D. R. Woodfield. 2002. Methanogenesis from forages fed to sheep. Proc. N. Z. Grassland Assoc. 64:121–125. doi:10.33584/jnzg.2002.64.2462

[CIT0070] Wang, L., D. Wang, Z. He, G. Liu, and K. C. Hodgkinson. 2010. Mechanisms linking plant species richness to foraging of a large herbivore. J. Appl. Ecol. 47:868–875. doi:10.1111/j.1365-2664.2010.01837.x

[CIT0071] Williams, S. R. O., P. J. Moate, M. H. Deighton, M. C. Hannah, W. J. Wales, and J. L. Jacobs. 2016. Milk production and composition, and methane emissions from dairy cows fed lucerne hay with forage brassica or chicory. Anim. Prod. Sci. 56:304–311. doi:10.1071/ANI5528

[CIT0072] Wong, C. C., and J. R. Wilson. 1980. Effects of shading on growth and nitrogen content of green panic and Siratro in pure and mixed swards defoliated at two frequencies. Aust. J. Agric. Res. 31:269–285. doi:10.1071/AR9800269

[CIT0075] Zubieta, A. S., J. V. Savian, W. D. S. Filho, M. O. Wallau, A. M. Gomez, J. Bindelle, O. J. F. Bonnet, and P. C. F. Carcalho. 2021. Does grazing management provide opportunities to mitigate methane emissions by ruminants in pastoral ecosystems?. Sci. Total Environ. 754:142029. doi:10.1016/j.scitotenv.2020.14029 33254863

